# Continuous Manipulation and Characterization of Colloidal Beads and Liposomes via Diffusiophoresis in Single- and Double-Junction Microchannels

**DOI:** 10.1021/acsnano.3c02154

**Published:** 2023-07-17

**Authors:** Adnan Chakra, Naval Singh, Goran T. Vladisavljević, François Nadal, Cécile Cottin-Bizonne, Christophe Pirat, Guido Bolognesi

**Affiliations:** † Department of Chemical Engineering, Loughborough University, Loughborough, LE11 3TU, United Kingdom; ‡ Department of Chemistry, University College London, London, WC1H 0AJ, United Kingdom; ¶ Manchester Centre for Nonlinear Dynamics, Department of Physics and Astronomy, University of Manchester, Manchester M13 9PL, United Kingdom; § Commissariat à l’Énergie Atomique, BP2, 33114, Le Barp, France; ∥ Institut Lumière Matière, UMR5306 Université Claude Bernard Lyon 1- CNRS, Université de Lyon, Villeurbanne Cedex, 69622, France

**Keywords:** diffusiophoresis, diffusio-osmosis, microfluidics, nanoparticles, liposomes, zeta potential

## Abstract

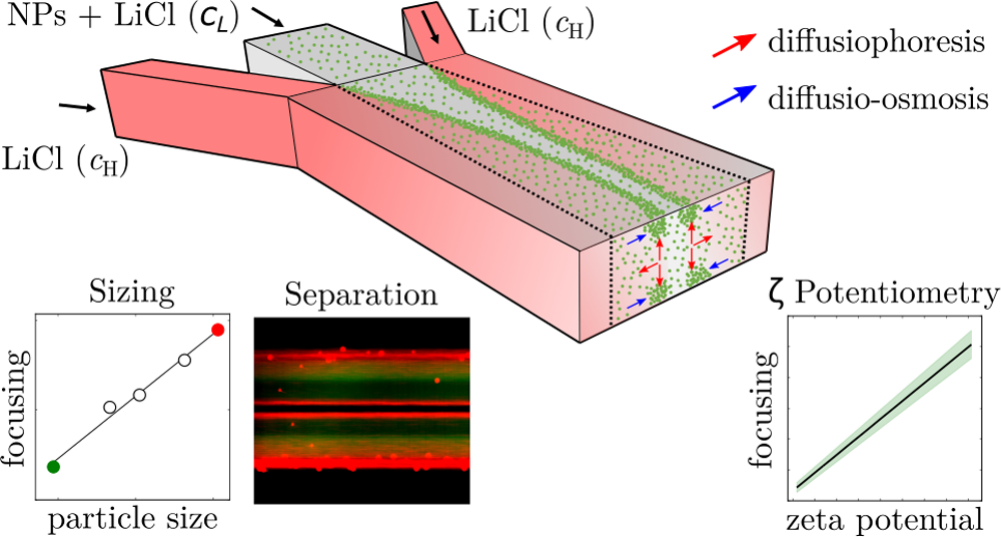

We reveal a physical mechanism that enables the preconcentration, sorting, and characterization of charged polystyrene nanobeads and liposomes dispersed in a continuous flow within a straight micron-sized channel. Initially, a single Ψ-junction microfluidic chip is used to generate a steady-state salt concentration gradient in the direction perpendicular to the flow. As a result, fluorescent nanobeads dispersed in the electrolyte solutions accumulate into symmetric regions of the channel, appearing as two distinct symmetric stripes when the channel is observed from the top via epi-fluorescence microscopy. Depending on the electrolyte flow configuration and, thus, the direction of the salt concentration gradient field, the fluorescent stripes get closer to or apart from each other as the distance from the inlet increases. Our numerical and experimental analysis shows that although nanoparticle diffusiophoresis and hydrodynamic effects are involved in the accumulation process, diffusio-osmosis along the top and bottom channel walls plays a crucial role in the observed particles dynamics. In addition, we developed a proof-of-concept double Ψ-junction microfluidic device that exploits this accumulation mechanism for the size-based separation and size detection of nanobeads as well as for the measurement of zeta potential and charged lipid composition of liposomes under continuous flow settings. This device is also used to investigate the effect of fluid-like or gel-like states of the lipid membranes on the liposome diffusiophoretic response. The proposed strategy for solute-driven manipulation and characterization of colloids has great potential for microfluidic bioanalytical testing applications, including bioparticle preconcentration, sorting, sensing, and analysis.

## Introduction

Synthetic and natural nanoparticles are ubiquitous in a wide range of chemical,^1^ bioanalytical,^2,3^ biomedical,^4−6^ and environmental^7^ applications. Control over nanoparticle motion is an important aspect for many of these applications, especially for bioanalysis, drug delivery, diagnostics, and environmental monitoring for which particle filtration, preconcentration, directed delivery, and purification are often required.^8−10^ Nanoparticle characterization in terms of size and surface properties, such as chemical composition and charge, is also key to several nanoparticle technologies. For example, particle size and zeta potential play a crucial role in many biological processes, like membrane-binding affinity, cellular uptake, and cytotoxicity.^11−14^ Size, surface charge, and surface composition also dictate the nanoparticles’ ability to penetrate through natural barriers, such as extracellular matrices^15,16^ and the blood brain barrier.^17^ Consequently, control over particle size and surface properties is essential for precision drug delivery applications, in which these properties are tuned to increase circulation time,^18,19^ achieve high therapeutic efficacy,^20,21^ and reduce toxicity.^22^ As a further example, extracellular vesicles, i.e., lipid vesicle naturally released and internalized by cells, have attracted much attention because of their potential as powerful therapeutic and diagnostic tools.^23^ Their size, surface charge, and biochemical composition reflect their biogenesis and determine the cellular uptake pathways used for intercellular communication.^24^ Thus, the characterization of the size and surface properties of these lipid vesicles is necessary to elucidate the many physiological and pathological processes with which they are involved,^25^ and to exploit their potential as drug carrier and disease biomarkers.

In the last two decades, there has been a growing interest in microfluidic strategies for both particle manipulation^26,27^ and characterization.^28,29^ This is due to the many advantages offered by microfluidic technologies compared to their conventional counterparts, including cost-effectiveness, reduced consumption of samples and reagents, high precision, portability, and capability to perform multiplexed analysis and continuous flow processing.^30−32^ In this context, diffusiophoresis—namely, the phoretic transport of colloidal particles induced by a solute concentration gradient—has emerged as a valuable tool for particle manipulation and characterization in microfluidic chips,^33,34^ and many devices have been proposed to exploit it for targeted delivery,^35,36^ focusing,^37^ trapping,^38,39^ accumulation,^40^ sorting, filtration, and separation.^41−45^ Furthermore, since the diffusiophoresis mobility of colloids can depend on particle size^35,41,46^ and surface charge,^47−49^ this transport mechanism has been exploited also for the detection and characterization of these particle properties. A low-cost zeta potentiometry microchip was developed by relying on the fluid and particle motion induced within dead-end microchannels via transient salt concentration gradients.^49^ This microfluidic device has a simple geometry and is cheap and easy to fabricate. However, it allows only for batch (i.e., noncontinuous) measurements, due to the transient nature of the salt concentration gradient and the need for regular flushing of the dead-end pores to replace the sample and prevent clogging.

Imposing a salt concentration gradient in a microchip also results in a diffusio-osmotic slip velocity at the charged walls of the fluidic channels.^50,51^ Such a slip velocity is typically weak and usually has negligible effects in pressure-driven flows within open micron-sized channels. Conversely, diffusio-osmotic effects can become significant in dead-end channels^35,52^ or in highly confined flows within nanotubes^53^ and nanochannels.^54^ Recently, diffusiophoresis and diffusio-osmosis have been jointly exploited in a cleverly designed microfluidic platform for the separation and characterization of liposomes and extracellular vesicles.^46^ In this device, a shallow tapered open-ended nanochannel is exposed to a steady salt concentration gradient, and this leads to the size- and charge-dependent accumulation of lipid vesicles nearby the region where the diffusio-osmotic flow velocity and the particle diffusiophoretic velocity balance each other. This microfluidic system enables the preconcentration of lipid vesicles as well as the accurate measurements of their diameter and zeta potential. Although this is the first significant exploitation of diffusio-osmotic flows for particle characterization, the sample analysis is again performed in batches, with a small fraction of the sample injected into the device being analyzed at any given time. Moreover, the use of nanochannels makes the device fabrication process more complex and expensive, because it requires costly cleanroom fabrication procedures such as reactive ion etching techniques.^46,51,54^ Nanochannel devices are also more vulnerable to obstruction and clogging compared to microchannel chips.

To address these limitations, we propose a microfluidic strategy for the manipulation and characterization of colloidal particles via solute concentration gradients in continuous flow, low-cost, and easy to fabricate microfluidic devices. First, we report and investigate a particle focusing mechanism occurring in a single Ψ-junction microchannel device. The intertwined effects of particle diffusiophoresis and flow diffusio-osmosis, induced by steady salt concentration gradients, enable the accumulation of colloidal beads and liposomes at multiple focusing regions nearby the charged walls of the microchannels. Notably, unlike previous studies, here the diffusiophoresis of nanoparticles and diffusio-osmotic flows along the wall do not compete one against the other by pushing particles along parallel and opposite directions, but instead, they act synergistically by moving particles along two perpendicular directions. This allows for the continuous and high-throughput manipulation of colloids in an open-ended micrometer-sized channel via diffusio-osmotic flows without resorting to dead-end or nanosized channel geometries, therefore reducing the risk of device clogging and avoiding the need of expensive device fabrication procedures. To exploit this mechanism, we introduce a double-junction device that can be used for the continuous separation and characterization of nanoparticles based on their size or zeta potential. Furthermore, we investigate how the chemical composition and lipid phase of the membrane affect the liposome diffusiophoretic response and establish a relationship between the latter and the charged lipid content of the membrane.

## Results and Discussion

### Particle Focusing in Single Junction Devices

**Figure 1:**
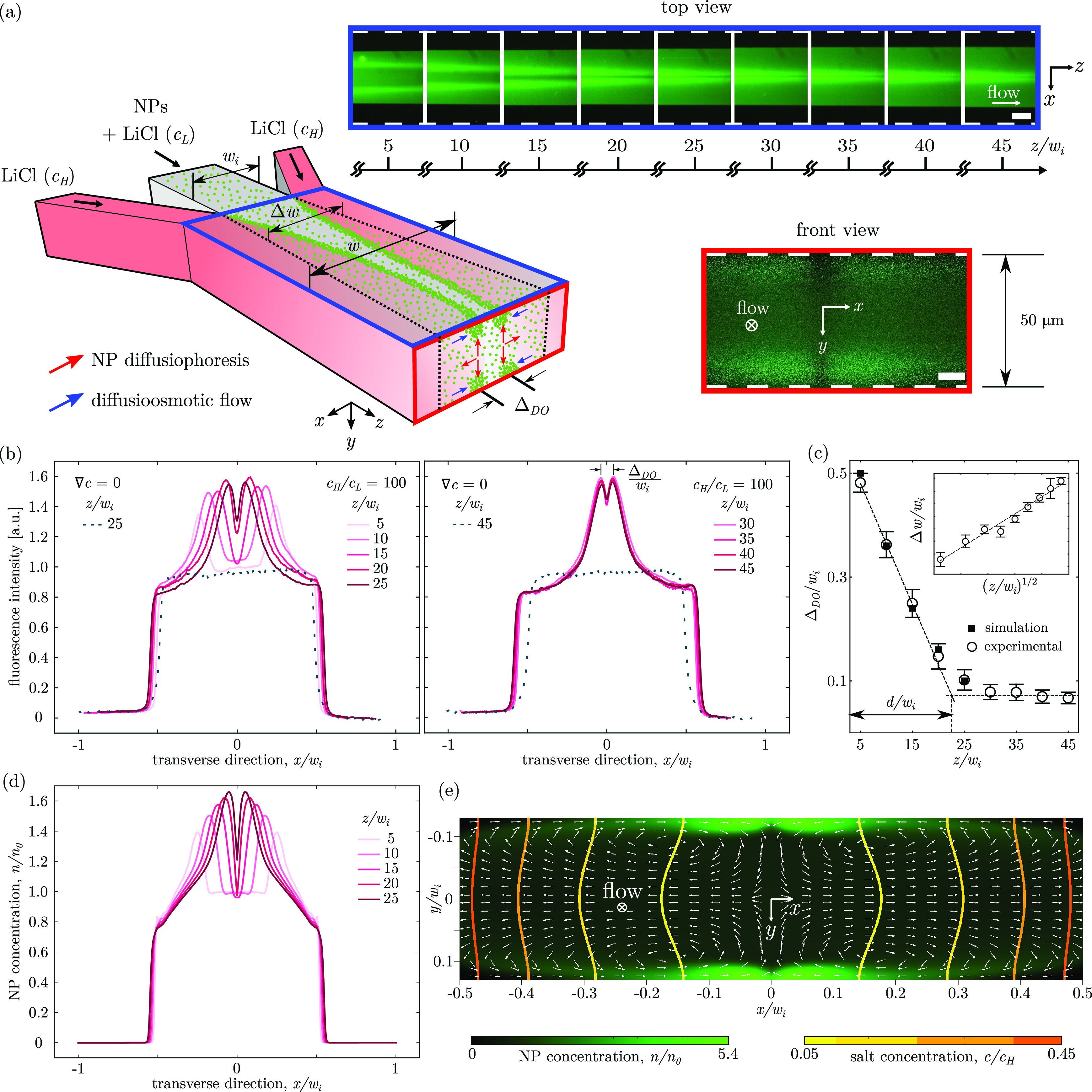
(a) Schematic and micrographs of a single Ψ-junction device. Outer streams: LiCl solution at a high concentration (*c*_*H*_). Inner stream: LiCl solution at low concentration (*c*_*L*_) seeded with fluorescent (*d* = 216.5 ± 0.9 nm) carboxylate polystyrene nanoparticles (NPs), shown as green dots. The decay of the distance Δ_DO_ between the accumulation peaks with the distance from the junction (*z*) is visible in the top view epi-fluorescence micrographs (blue rectangle). The micrographs were acquired with the focal plane located near the glass (bottom) wall of the device. The four accumulation regions in the transverse (*x*, *y*)-plane are visible in the confocal image of the channel cross-section at a distance of *z*/*w*_*i*_ = 25 from the junction (front view, red rectangle). (b) Experimental intensity profiles along the transverse *x*-direction with a salt gradient (solid lines) and without a salt gradient (dashed lines) at increasing distances *z*/*w*_*i*_. (c) Experimental and numerical peak separation distance Δ_DO_ as a function of distance *z*/*w*_*i*_. Inset shows the profile of the colloidal bandwidth, Δ*w*, as a function of the distance *z*/*w*_*i*_. (d) *y*-Averaged NP concentration profiles along the transverse *x*-direction at increasing distances *z*/*w*_*i*_, predicted by numerical simulations. (e) Numerical map of the channel cross-section at a distance *z*/*w*_*i*_ = 25 showing the NP concentration field, the salt concentration isolines, and the total particle velocity field, ***u***_p_ = ***u*** + ***u***_DP_, streamlines (white arrows) with diffusio-osmotic slip velocity, ***u***_DO_, at the channel walls.

A Ψ-junction microfluidic device was used to generate a steady-state gradient of salt concentration (referred to as *c* in the following) by pumping a low concentration (*c*_*L*_ = 0.1 mM) of LiCl aqueous solution in the inner channel and a high concentration (*c*_*H*_ = 10 mM) of LiCl aqueous solution in the outer channels (Figure 1a). A similar flow configuration was adopted in previous studies^37−39,55,56^ to investigate the dynamics of charged colloids under steady-state salt concentration gradients and continuous flow conditions.

In a first set of experiments, carboxylate polystyrene nanoparticles (NPs), 216.5 ± 0.9 nm in diameter (Invitrogen, USA) and ζ = −54.9 ± 0.7 mV, are dispersed in the inner flow only, thus leading to the formation of a fluorescent stream of colloidal solution, hereby referred to as a colloidal band, within the central region of the channel. The boundaries of the colloidal band are represented by the black dotted lines in the 3D schematic of the device in Figure 1a. An *x*, *y*, and *z*-axis reference system is introduced as shown in the figure. The origin of the *z*-axis is located at the junction, whereas the origins of the *x*- and *y*-axes are located at the midpoints along the channel width and depth, respectively. The parallel streams of electrolyte solutions at different salt concentrations generate a salinity gradient in the direction transverse to the flow (red arrows parallel to the *x*-direction in Figure 1a), which causes the broadening of the colloidal band due to the diffusiophoresis migration of charged nanoparticles toward higher salt concentration regions. In agreement with previous studies,^37,56^ the particle transverse profiles show an enhanced diffusive dynamics in the transverse direction, and the colloidal bandwidth, Δ*w*, increases linearly with the square root of the longitudinal distance *z* from the junction (inset in Figure 1c). As discussed by Abecassis and co-workers, the enhanced colloid diffusivity in the transverse direction is caused by the coupling of the diffusiophoretic migration of particles with the underlying salt diffusion process.^37^

The chemical gradient field also prompts the appearance of two distinct peaks of focused nanoparticles (when looking at the channel from above), as shown by the epi-fluorescence images in the blue rectangle of Figure 1a. The peaks are separated from each other by a distance Δ_DO_, initially equal to the inner channel width *w*_*i*_. The value of Δ_DO_ rapidly decreases with *z* over a typical distance *d* ≃ 23*w*_*i*_ and eventually plateaus at a constant *saturation* value (Figure 1c). The decay distance *d* is estimated by intersecting two straight lines fitting the Δ_DO_ versus *z* plot for *z*/*w*_*i*_ → 0 and *z*/*w*_*i*_ → 45, respectively. Particle peaks do not form when no salt concentration gradient is imposed (Figure S1b,c in Supporting Information). The particle focusing effect is quantified by plotting the fluorescence intensity profile against the transverse *x*-direction (perpendicular to the direction of the flow) at different distances, *z*/*w*_*i*_, downstream of the junction (Figure 1b). The profiles are normalized with respect to the intensity of the colloidal solution with the particle concentration, *n*_0_, injected in the inner channel of the device. Note that the epi-fluorescence micrographs are generated from the convolution of the particle fluorescence intensity with the microscope point-spread function.^57^ Thus, the intensity profiles of Figure 1b are the result of an integration of the particle fluorescence intensity over an optical window, whose characteristic size in the vertical *y*-direction is on the order of the depth of field of the optical system (ca. 10 μm). For the micrographs in Figure 1a, the midpoint of this optical window, which coincides with the focal plane of the microscope objective, was located near the bottom glass wall of the device. Note that epi-fluorescence images were also acquired with the focal plane located near the top PDMS wall and at the midpoint along the depth of the microchannel, but no significant change in the micrographs and corresponding intensity profiles could be detected, as shown in Figure S3 in the Supporting Information. For this reason, all epi-fluorescence micrographs and intensity profiles reported in the paper were acquired with a focal plane located near the bottom glass wall, unless otherwise stated. From the intensity profiles in Figure 1b, we can observe a clear effect of focusing of the nanoparticles (solid lines) when compared to the case without a salinity gradient (dashed lines). A confocal scan of the (*x*, *y*)-plane confirms that under salinity gradient conditions charged particles are accumulating at the top and bottom walls of the device in four symmetrical positions (red rectangle in Figure 1a). In the absence of a salinity gradient, the particle distribution profile in the same plane is uniform (Figure S1c in Supporting Information), therefore the observed particle dynamics is driven by the salt contrast generated in the microchannel. Interestingly, the confocal image in Figure 1a shows slight asymmetry in the peak intensity and peak separation distance between the two walls of the device. Specifically, the peaks nearby the bottom glass wall are slightly more intense and close to each other than the peaks nearby the PDMS wall. This asymmetry is possibly due to the material differences between the two surfaces, as explained later in this section.

The formation of peaks at the channel walls are in agreement with the observations reported in our previous study, where we investigated the diffusiophoresis manipulation of charged nanoparticles in a Ψ-junction channel fitted with a microgrooved substrate.^38^ Our analysis revealed that the particle accumulation regions at the flat wall of the device are induced by the vertical component of the salt concentration gradient which is originated by the Poiseuille-like velocity profile in the rectangular microchannel.^38^ Conversely, the convergence of the peaks in the (*z*, *x*)-plane toward lower salinity regions is unexpected. Indeed this particle behavior seems to contradict the well-established interpretation of particle diffusiophoresis,^50^ according to which colloids migrate toward higher salinity regions with a diffusiophoresis velocity ***u***_DP_ = Γ_DP_**∇**(ln *c*) when the diffusiophoresis mobility Γ_DP_ is positive—as is the case for negatively charged particles in LiCl solutions.^37^ In fact, the coefficient Γ_DP_ can be expressed as the contribution of a chemiphoretic term, which is always positive, and an electrophoretic term, the sign of which is given by the product βζ, where , and *D*_+_ and *D*_–_ are the diffusivities of the salt cations and anions, respectively. For LiCl, β < 0 and, thus, for negatively charged nanoparticles (ζ < 0), the electrophoretic term is also positive and Γ_DP_ > 0. For the same reason, the diffusio-osmosis mobility Γ_DO_ of a negatively charged surface, such as a PDMS or glass wall, in a LiCl solution is also positive, and the diffusio-osmotic slip velocity at the channel walls, which can be expressed as ***u***_DO_ = −Γ_DO_**∇**(ln *c*), has an opposite direction to the one of particle diffusiophoresis. Consequently, the observed particle migration toward lower salinity regions suggests that additional phenomena, such as hydrodynamics and diffusio-osmosis induced flows, must be involved in the mechanism responsible for the transverse deviation of the peaks.

**Figure 2:**
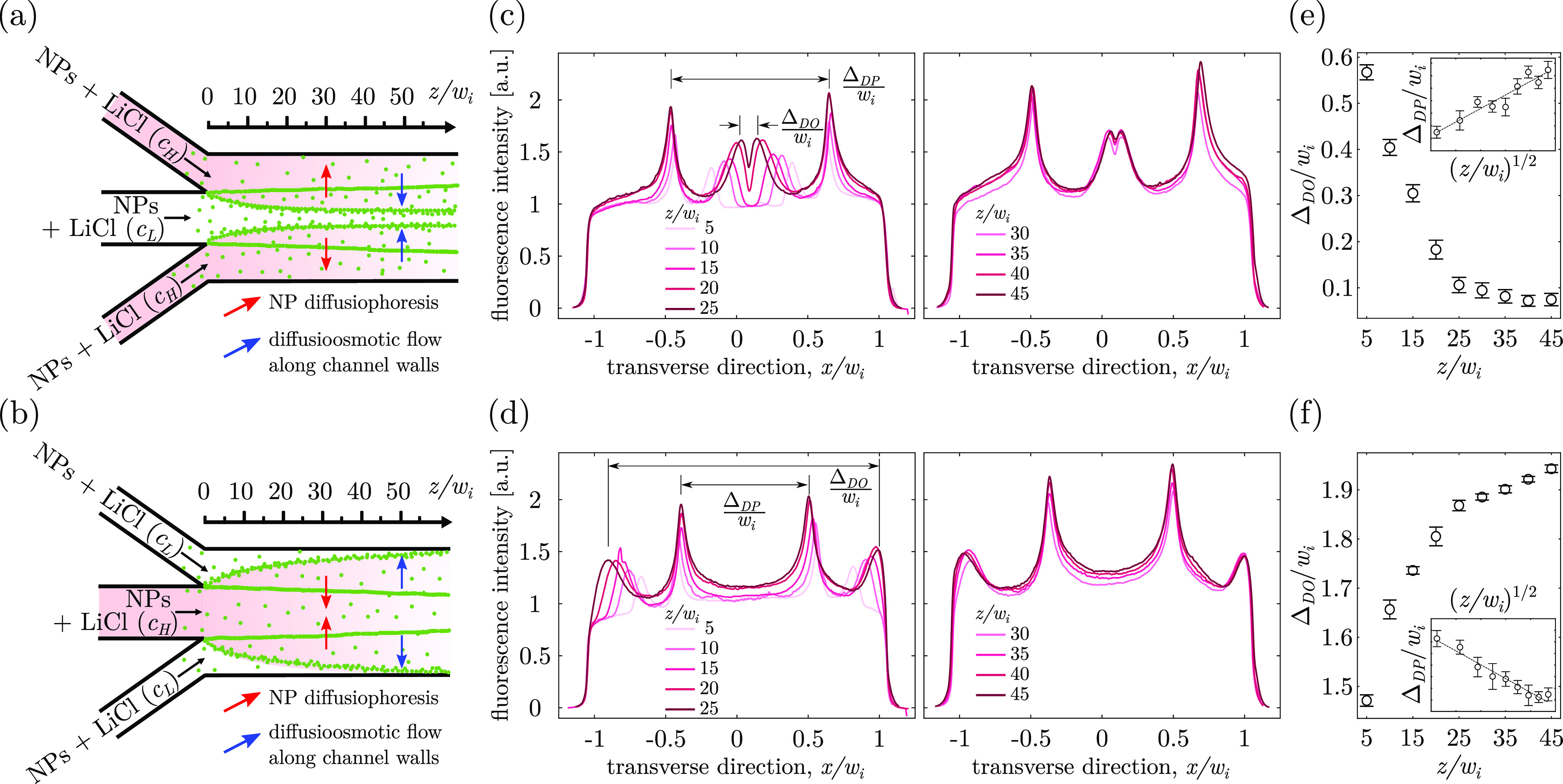
Relationship between the direction of the salt gradient and the dynamics of the peak displacement mechanism due to diffusio-osmosis along the top and bottom channel walls. (a,b) Schematic diagrams of a single Ψ-junction microchip, where carboxylated NPs are uniformly injected (in all channels) with two different configurations of salt gradient. In (a), high concentration salt is in the outer channels (*c*_*H*_/*c*_*L*_ = 100), while in (b) high salt concentration is in the inner channel. Red-to-white shade qualitatively indicates the field of salt concentration. Red (blue) arrows show the direction of diffusiophoresis transport (diffusio-osmosis flow along the channel walls). (c,d) Normalized fluorescent intensity profile plots along the transverse direction at various distances *z*/*w*_*i*_ downstream of the junction, for the configurations depicted in schematics (a) and (b), respectively. (e,f) Normalized longitudinal distance Δ_DO_ plotted with respect to the normalized distance *z*/*w*_*i*_ for the configurations (a) and (b), respectively. The corresponding plots Δ_DP_ vs (*z*/*w*_*i*_)^1/2^ for the two configurations are shown in the insets.

To verify our hypothesis, two additional experiments with different flow configurations were conducted (Figure 2a,b). First, a salinity gradient was imposed as done in the experiment depicted above (high salt concentration *c*_*H*_ in the outer channels and low concentration *c*_*L*_ in the inner channel). However, contrary to what was done in the initial experiment, a homogeneous solution of nanoparticles was injected into both the inner and outer channels (Figure 2a). In a second configuration, the chemical gradient was swapped (high salt concentration in the inner channel; Figure 2b). As shown by the fluorescence intensity profiles along the transverse direction (Figure 2c,d), the focused peaks induced by diffusiophoresis effects converge (Δ_DO_ decreases with the distance) in the first configuration (Figure 2a) and diverge (Δ_DO_ increases with the distance) in the second configuration (Figure 2b). As observed in the previous experiment, the variation of Δ_DO_ with *z* (Figure 2e,f) is rapid at shorter distances from the junction (*z*/*w*_*i*_ ≲ 25) and slower at larger distances (*z*/*w*_*i*_ ≳ 25). By swapping the salinity gradient, we effectively interchanged the chemically generated electrical field and pressure gradient that give birth to the fluid flow near the charged walls induced by the diffusio-osmosis slip velocity, ***u***_DO_ = −Γ_DO_**∇**(ln *c*), where Γ_DO_ has the same sign and order of magnitude as Γ_DP_. No physical parameters or properties in our system were varied otherwise. Consequently, the systematic counterintuitive transversal behavior of focused peaks in both configurations (Figure 2e,f) suggests that they were deeply affected by diffusio-osmosis flows along the channel walls, which also justifies our choice of notation, Δ_DO_, for the peak distance. In other words, the peak displacement is always against the diffusiophoresis-driven motion predicted for Γ_DP_ > 0 (red arrows in Figure 2a,b), and, instead, it has always the same direction of the diffusio-osmosis slip velocity for Γ_DO_ > 0 (blue arrows in Figure 2a,b). Note that in these two complementary experiments the presence of particles in both inner and outer streams results in the formation of two additional but narrower and more intense peaks separated from each other by a distance Δ_DP_ (Figure 2c,d) which evolves in an opposite way compared to Δ_DO_ (insets in Figure 2e,f). These peaks had been previously reported^56^ and are intrinsically different from the ones discussed so far. Indeed, they form as the transverse *x*-component of the chemical gradient induces a particle migration toward higher salt regions via diffusiophoresis only, thus the notation Δ_DP_. This causes nanoparticles to accumulate along the entire depth of the channel and not just nearby the channel walls.^56^ In addition, the transversal migration of these peaks is extremely slow, as their separation distance Δ_DP_ is proportional to *z*^1/2^, which is notably the same scaling as Δ*w*. Conversely, the separation distance Δ_DO_ changes much faster with the distance from the junction (Figure 2e,f).

A numerical analysis was performed in COMSOL Multiphysics to confirm our interpretation of the experimental observations. The numerical hydrodynamic velocity field ***u***, salt concentration *c*, and particle concentration *n* were calculated in a 3D domain consisting of a straight rectangular channel. A slip velocity ***u***_DO_ = −Γ_DO_**∇**(ln *c*) was imposed at the channel walls and the particle velocity ***u***_p_ was calculated as the sum of the hydrodynamic velocity and the diffusiophoresis velocity, ***u***_p_ = ***u*** + ***u***_DP_. The value of Γ_DO_ for the channel walls could not be measured, so it was used as an adjusting parameter in the model to achieve a good match between experimental and numerical results (see the Numerical Methods section for details). The simulated transverse profiles of the normalized particle concentration, *n*/*n*_0_, at increasing distances *z* downstream of the junction, are shown in Figure 1d, and they are in good agreement with the fluorescence intensity profiles measured in the experiments (Figure 1b). It is worth noting that a close quantitative match is not expected since the experimental profiles correspond to the convolution of the particle fluorescence intensity with the microscope point-spread function,^57^ whereas the numerical profiles are directly obtained from the simulated particle concentration field by averaging the concentration over the channel depth (*y* direction). A good quantitative agreement between experiments and simulations can be seen for peak separation Δ_DO_ at increasing distances from the junction (Figure 1c), which is consistent with the fact that the effect of the microscope point-spread function on Δ_DO_ measurements should be negligible. Figure 1e shows the simulated particle concentration field on the plane perpendicular to the flow direction at *z*/*w*_*i*_ = 25, and this is in good agreement with the confocal image of the channel cross-section at the same distance from the junction (Figure 1a). As expected, the salt concentration isolines in the inner region of the channel (|*x*|/*w*_*i*_ < 0.5), shown in Figure 1e, are bent toward the outer flow because of the Poiseuille-like hydrodynamic velocity profile. Consequently, the onset of a vertical component of the salt concentration gradient and, thus, of the diffusiophoresis velocity causes the accumulation of particles at the top and bottom walls. The diffusio-osmosis slip velocity at the walls induces the formation of four symmetric recirculation regions in the in-plane total particle velocity field ***u***_p_, whose streamlines are shown by white arrows in Figure 1e. As a result, the accumulated particles are advected along the top and bottom walls toward the central region of the channel, namely, from higher to lower salt concentration regions. To summarize, the observed particle behavior is governed by a combination of particle diffusiophoresis along the vertical axis, which induces particle accumulation, and diffusio-osmosis flow along the horizontal walls, which pushes the accumulation peak toward the center of the channel. It is worth noting that this interpretation is consistent with the slight asymmetry in the particle distribution observed in the confocal image of Figure 1a. Indeed, the larger diffusio-osmosis mobility expected for the glass surface compared to the PDMS surface (see the estimates in SI), should lead to shorter peak separation distances at the bottom glass wall, as shown in the confocal image of Figure 1a. Similarly, the intensity of the accumulation peaks may depend also on the diffusio-osmosis mobility and, hence, on the material properties of the two surfaces, thus justifying the noticeable slight difference in the intensities of the particle accumulation peaks between the two walls.

### Particle Size Detection and Size-Based Separation in Double-Junction Devices

**Figure 3:**
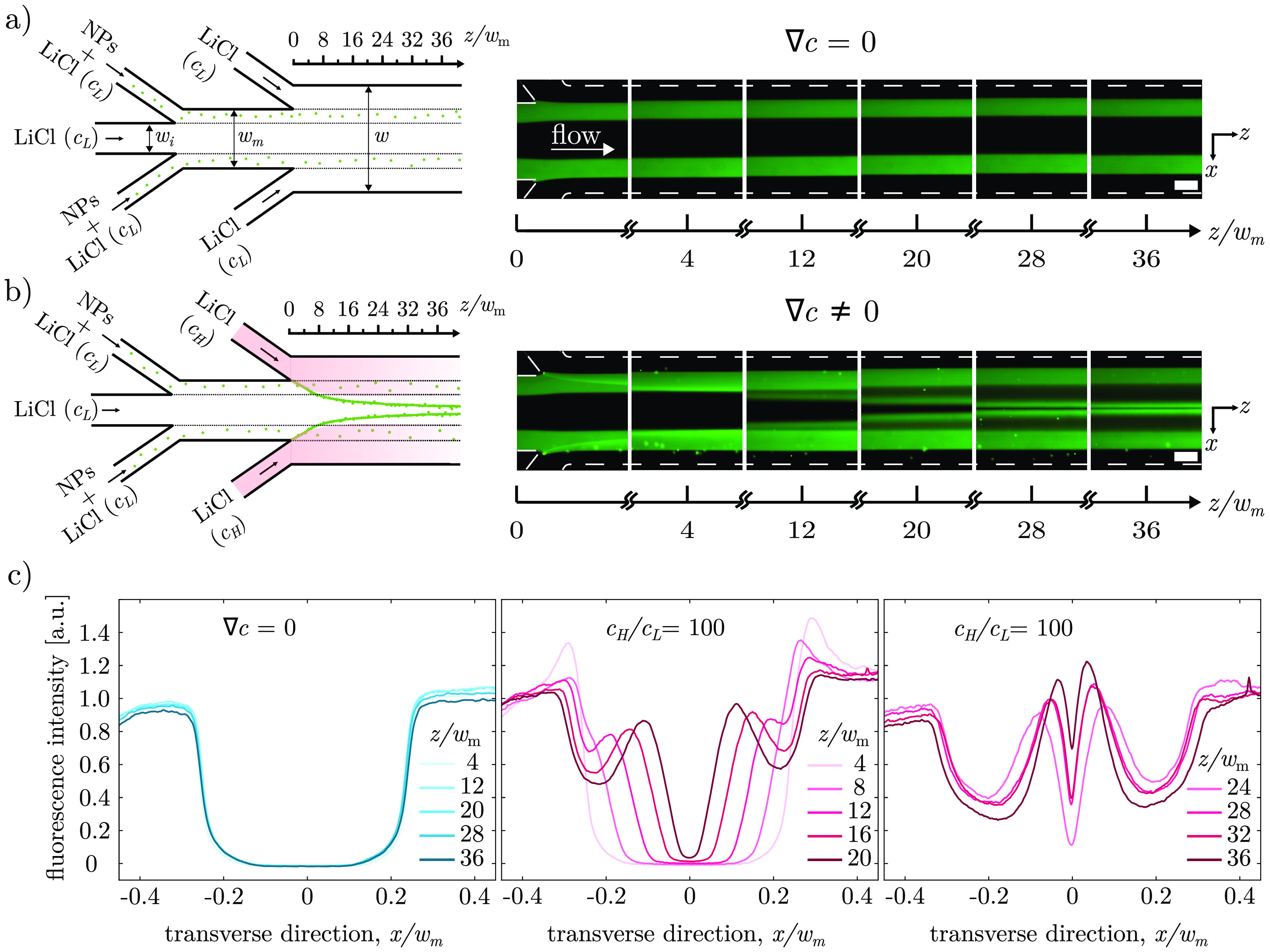
Particle focusing and fractioning in a double Ψ-junction device. (a,b) Schematic diagrams of the double-junction microchip and the corresponding epi-fluorescence micrographs showing the fluorescent carboxylate polystyrene nanoparticles (*d* = 549.8 ± 6.8 nm, ζ = −50.4 ± 0.2 mV) at various distances from the junction when there is no salinity gradient (**∇***c* = 0) in (a) and when there is a salinity gradient (*c*_*H*_/*c*_*L*_ = 100) in (b). The widths of the inner inlet channel, the middle channel, and the main channel are *w*_*i*_, *w*_*m*_, and *w*, respectively. Scale bar is 75 μm. (c) Normalized fluorescent intensity profiles when there is no salinity gradient (blue curve) and when there is a salinity gradient (red curve) at various distances downstream of the junction.

The Ψ-junction microchip, depicted in Figure 1a, could be potentially adopted for the online preconcentration, via solute-driven transport, of charged synthetic or biological colloids, including macromolecules,^58^ liposomes,^35^ exosomes,^46^ viruses,^59^ and bacterial cells.^60^ This can be particularly useful for applications such as microfluidic point-of-care diagnostics and point-of-need bioanalytical testing, provided that the target analytes are charged and thus susceptible to diffusiophoresis migration. Alternatively, the same device could be used for the solute-driven accumulation of charged nanoparticles that are conjugated to recognition moieties, such as antibodies or aptamers, for the capture and detection of the target molecules.^61^ Furthermore, since the diffusiophoresis mobility depends on both particle size and zeta potential,^35,47^ the diffusiophoresis-driven accumulation of nanoparticles at the device walls could be exploited also for particle characterization, fractioning (commonly known as field flow fractioning) and sorting. However, the device configuration shown in Figure 1 does not lend itself to such applications, because the accumulation peaks are advected toward the central region of the channel, thus overlapping with the bulk colloidal stream. Consequently, particle fractioning and sorting are not possible. Moreover, the fluorescence intensity of the accumulation peaks is partially screened by the background fluorescence signal generated from the colloids in the bulk (Figure 1a,b), thus limiting the accuracy of the peak intensity detection and hampering the ability to characterize particles by charge or size. These limitations could be overcome if the accumulation peaks migrated away from the bulk colloidal stream. To achieve this, first we tested a different flow configuration, whereby a salinity gradient was imposed as done in the experiment of Figure 1—namely, higher salt concentration *c*_*H*_ in the outer channels and lower salt concentration *c*_*L*_ in the inner channel, but the colloidal particles were present in the outer stream only. However, this test came to no avail (see Figure S2 in Supporting Information), since the accumulation peaks did not form. This is because the salt concentration isolines, shown in Figure 1e, are bent outward only within the inner stream region of the channel (|*x*|/*w*_*i*_ ≤ 0.5), but no colloids are now present in that region. As a result, the vertical component of the salt gradient no longer leads to the migration of colloids toward the top and bottom walls and the consequent formation of the particle accumulation peaks. Therefore, an alternative chip design was required to exploit the observed phenomenon of particle focusing for particle fractioning, separation, and characterization. Figure 3a,b depicts the blueprint of the double Ψ-junction microchip designed for this purpose. The channel geometry consists of a narrower (upstream) junction, where the inner inlet channel of width *w*_*i*_ meets the middle inlet channels and merges into the middle channel of width *w*_*m*_. This is followed by a wider (downstream) junction, where the middle channel meets the outer inlet channels and merges into the main channel of width *w*. The downstream junction is used to regulate the salt concentration gradient in the device by swapping the flow streams from the outer inlets between a low salt concentration (*c*_*L*_) solution, leading to no salt gradient (Figure 3a), and a high salt concentration (*c*_*H*_) solution, generating a steady-state salt gradient (Figure 3b). The upstream junction allows control of the position of the colloidal stream within the main channel. All streams injected from the inlet channels of the upstream junction have a low salt concentration (*c*_*L*_), but only the middle inlet streams are laden with colloidal particles. Consequently, the central region of the middle and main channels remains particle-free so that upon imposition of the salt concentration gradient the focused particle peaks can converge into this region without overlapping with the bulk colloidal stream.

The double-junction device was first tested using carboxylate polystyrene nanoparticles (*d* = 549.8 ± 6.8 nm, ζ = −50.4 ± 0.2 mV). Epi-fluorescence micrographs were taken at different distances downstream of the junction, *z*/*w*_*m*_, in the absence or presence of a salt concentration gradient (Figure 3a,b). Note that for the double-junction device, the width of the middle channel, *w*_*m*_ = 250 μm, is used as the characteristic channel size, and all lengths are normalized with respect to it. The corresponding fluorescent intensity profiles along the transverse direction *x* show that peak formation occurs when a salinity gradient is imposed (red curves in Figure 3c). Crucially, the peaks move toward the central region of the channel, which would otherwise be empty in the absence of a salt concentration gradient (blue curves, Figure 3c).

**Figure 4:**
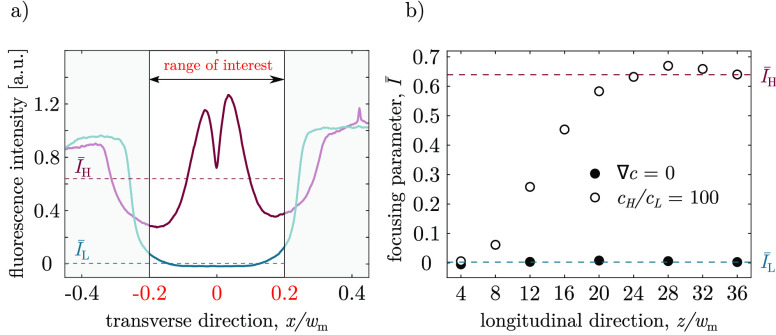
Focusing parameter in double-junction device for carboxylate polystyrene nanoparticles (*d* = 549.8 ± 6.8 nm, ζ= −50.4 ± 0.2 mV) (a) Normalized fluorescent intensity profile at *z*/*w*_*m*_ = 36 without a salinity gradient (blue curve) and with a salinity gradient (red curve). The range of interest corresponds to a selected central region of the channel, *x*/*w*_*m*_ ∈ [−0.2, 0.2]. The focusing parameter, *I̅*, is calculated as the average fluorescence intensity over the range of interest. (b) Focusing parameter at different distances downstream of the junction (*z*/*w*_*m*_) under no salinity gradients (solid circles) and with a salinity gradient (empty circles). The blue and red dashed lines are the focusing parameter values corresponding to their respective intensity plots in (a) at *z*/*w*_*m*_ = 36.

To quantify this focusing effect, we introduce the focusing parameter, *I̅*, defined as the average value of the normalized fluorescent intensity profile within the range of interest, *x*/*w*_*m*_ ∈ [−0.2, 0.2], which is equivalent to a transverse section, ca. 50 μm wide, located at the center of the channel (Figure 4a). Note that the width of the range of interest is chosen to exclude the fluorescence intensity generated by the particles in the bulk colloidal stream, namely, the gray shaded regions in Figure 4a. Figure 4b shows the focusing parameter at increasing distances *z*/*w*_*m*_ downstream of the junction in the presence (empty circles) and absence (solid circles) of a salt concentration gradient. Under the examined conditions, at *z*/*w*_*m*_ = 36, the focusing parameters are *I̅*_*H*_ = 0.66 and *I̅*_*L*_ = 0.03 with and without a salt concentration gradient, respectively.

**Figure 5:**
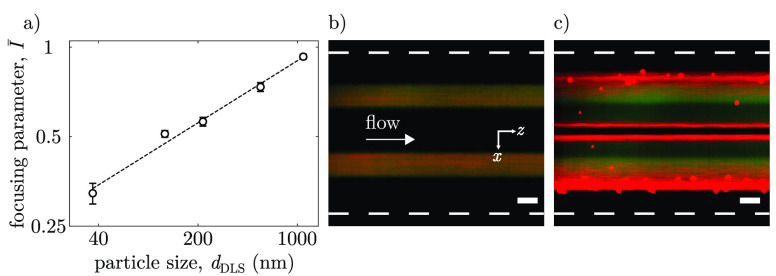
Particle size characterization and particle separation. (a) Average focusing parameters for different particle sizes at approximately the same zeta potential (see Table 1). (b-c) Epi-fluorescence images taken at *z*/*w*_*m*_ = 36 showing particle dynamics of mixed yellow green fluorescent (505/515) 36 nm and red fluorescent (580/605) 1098 nm carboxylate polystyrene colloids under no salinity gradient (b) and with a salinity gradient (c). Scale bar: 50 μm. White dashed line corresponds to the microfluidic channel boundaries.

Since the formation of the accumulation peaks is driven by the diffusiophoresis migration of charged nanoparticles from the channel bulk toward the top and bottom walls of the microchannels, it is reasonable to expect that the peak intensity, and thus the focusing parameter, can be correlated to the diffusiophoresis coefficient of the nanoparticles. For a charged nanoparticle in an electrolyte solution, the diffusiophoresis coefficient Γ_DP_ is an increasing function of the particle size,^35,47^ if the thickness of the Debye layer κ^–1^ formed around the particle is a few percent greater than the particle’s radius *a*, namely (*κa*)^−1^ ≳ 1%. On the other hand, for (*κa*)^−1^ → 0, the coefficient Γ_DP_ levels off to a constant value that is independent of the particle size. In a 0.1 mM LiCl solution, the Debye layer thickness is κ^–1^ = 32 nm, therefore it is expected that for submicron particles (2*a* ≲ 1 μm and (*κa*)^−1^ > 6%) the coefficient Γ_DP_ and the accumulation peak intensity increase with the particle size. To verify this hypothesis, we measured the focusing parameter for carboxylate polystyrene particles with a diameter ranging from tens of nanometers to one micrometer. Note that the zeta potentials of the particles are very similar for all diameters (Table 1). From the logarithmic plot in Figure 5a, it is apparent that the focusing parameter *I̅* increases with the particle diameter, *d*_*DLS*_, determined via dynamic light scattering (DLS). Interestingly, log *I̅* and log *d*_*DLS*_ are linearly correlated (*R*^2^ = 0.99) as follows
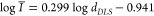
1where the value of *d*_*DLS*_ is expressed in nanometers. Consequently, eq 1 can be effectively used as a calibration line for the microfluidic characterization of the size of nanoparticles of similar zeta potential, whereby the focusing parameter *I̅* is measured experimentally to determine the nanoparticle diameter as follows
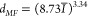
2with the value of the microfluidically measured diameter, *d*_*MF*_, expressed in nanometers. The uncertainty σ_*d*_ on *d*_*MF*_ can be calculated from the experimental uncertainty σ_*I*_ on *I̅* via error propagation in eq 2, which leads to σ_*d*_^2^ = 11.18*d*_*MF*_^2^σ_*I*_^2^/*I̅*^2^. Table 1 shows a comparison between the particle diameter, *d*_*DLS*_, measured via DLS, and the particle diameter, *d*_*MF*_, measured via the proposed microfluidic method. From the absolute values of the relative difference between the DLS and microfluidic measurements, ε_*r*_ = |*d*_*DLS*_ – *d*_*MF*_|/*d*_*DLS*_, it can be concluded that our microfluidic device can be successfully used to measure the diameter of submicron particles within a reasonable accuracy.

**Table 1:** Comparison between Dynamic Light Scattering (DLS) and Microfluidic (MF) Characterization of Submicron Particle Size^a^

*I̅*	*d*_*DLS*_ (nm)	ζ (mV)	*d*_*MF*_ (nm)	ε_*r*_
0.323 ± 0.03	36 ± 0.2	–58.3 ± 1.4	32 ± 9	12%
0.511 ± 0.01	117 ± 0.5	–57.6 ± 1.2	150 ± 13	28%
0.561 ± 0.02	217 ± 0.9	–54.9 ± 0.7	206 ± 23	5%
0.735 ± 0.03	550 ± 6.8	–50.4 ± 0.2	505 ± 57	8%
0.929 ± 0.02	1098 ± 29	–55.5 ± 0.7	1108 ± 70	1%

a *I̅* is the measured focusing parameter, *d*_*DLS*_ is the particle size determined via DLS, ζ is the particle zeta potential, *d*_*MF*_ is the particle size determined microfluidically via eq 2, and ε_*r*_ is the absolute value of the relative difference between *d*_*DLS*_ and *d*_*MF*_. The error for *d*_*DLS*_ and ζ represents the standard deviation obtained from the instrument. The error for *I̅* is the standard deviation from experimental mean values.

The combined effects of peak formation and drift toward the center of the channel can be exploited also for particle size-based separation. To demonstrate this additional application of the double-junction chip, a binary colloidal mixture of 36 nm diameter and 1.098 μm diameter particles was injected into the device. The carboxylate polystyrene particles were stained with fluorophores with different emission peaks, namely, 515 nm (yellow-green) for the smaller particles and 605 nm (red) for the larger particles. In the absence of a salt concentration gradient, both populations of particles are advected by the flow and remain confined in the same regions of the channel where they are initially injected (Figure 5b). Upon imposition of a salt contrast (*c*_*L*_ = 0.1 mM, *c*_*H*_ = 10 mM), the larger (red) particles are strongly focused at the center of the channel, where they form two intense peaks. Conversely, the smaller (yellow-green) particles do not form any accumulation peak and only a small fraction of them drift toward the center channel. Furthermore, the two colloidal bands made of larger particles, expand along the transverse *x*-direction toward the higher salt concentration (outer) regions of the channel due to diffusiophoresis,^37^ with two additional focusing peaks forming at these locations. On the other hand, the colloidal bands made of smaller particles do not drift toward the outer regions because of their smaller diffusiophoresis coefficient. As a result, diffusiophoresis and diffusio-osmosis can be effectively exploited for the microfluidic separation and sorting of the two populations of particles according to their size. It is worth mentioning that under the current experimental conditions, it is not possible to achieve high separation efficiencies for similarly sized particle populations. However, the relationship between the focusing parameter and the particle diameter depends on both the diffusiophoresis migration of particles and the diffusio-osmotic flows at the channel walls, which in turn can be adjusted by controlling the properties of the electrolyte solutions (e.g., ion type, ionic strength, pH) and the zeta potential of the channel walls. Finding an optimal combination of particle diffusiophoresis and wall diffusio-osmosis mobilities could be a potential strategy to achieve higher separation efficiencies for populations of similarly sized colloidal particles.

### Detection of Liposome Zeta Potential and Membrane Composition in Double-Junction Devices

The range of applications of the double-junction device can be expanded further by leveraging the dependence of the diffusiophoresis coefficient on the zeta potential, which is a key property of colloidal systems. To this end, we used nanosized unilamellar liposomes whose surface charge and, thus, zeta potential can be easily tuned by adjusting the lipid membrane composition. Such nanoparticles, which are often used as drug carriers^62,63^ and cell membrane models,^64^ are hence suitable for investigating the effect of zeta potential on the focusing phenomenon in the double-junction device.

**Figure 6:**
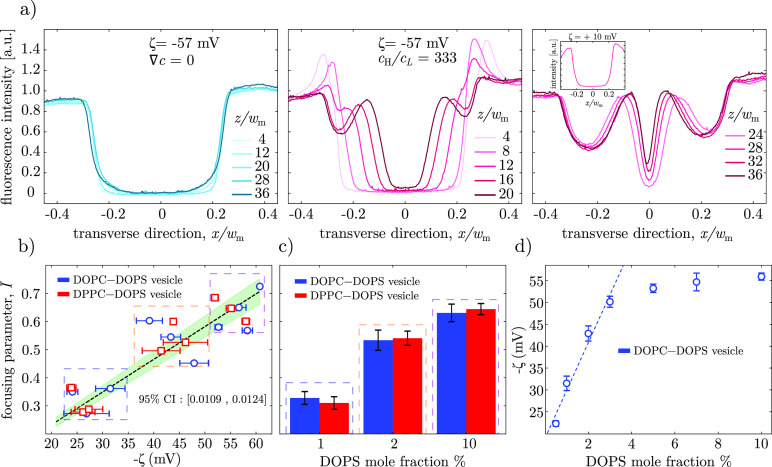
(a) Normalized fluorescent intensity profiles along the transverse direction *x* and at varying distances *z* from the downstream junction without (blue curves) and with (red curves) a salinity gradient for negatively charged (ζ = −57 mV) 10:90 DOPS:DPPC liposomes. Inset shows the fluorescent intensity profile for zwitterionic DPPC liposomes (ζ = +10 mV). (b) Experimental focusing parameter against zeta potential for DOPC (blue) and DPPC (red) based liposomes with varying anionic DOPS concentrations (navy box = 1% PS, orange = 2% PS, purple = 10% PS). The green shaded region represents the 95% confidence interval for the upper and lower bound values of the slope. (c) Experimental focusing parameter against the anionic DOPS lipid content. (d) Relation between the vesicle zeta potential and anionic DOPS lipid content for DOPC-DOPS vesicles. The dashed line corresponds to the linear regression of the first four data points. Fabricated liposomes had an average size of *d* = 166 ± 4 nm.

In the first set of experiments, we fabricated negatively charged liposomes (*d* = 166 ± 4 nm, ζ ≈ −57 mV, polydispersity index ≈ 0.1) by adding 10% mole fraction of the anionic 1,2-dioleoyl-*sn*-glycero-3-phospho-l-serine (DOPS) phospholipid to the zwitterionic 1,2-dipalmitoyl-*sn*-glycero-3-phosphocholine (DPPC) phospholipid. The phophoserine headgroup (PS) was specifically chosen as its presence in the outer leaflet of the membranes of biological particles can be associated with pathological and physiological processes, such as in tumor-derived exosomes^65^ and during cell apoptosis.^66^ The vesicles were dispersed in a low salt concentration (*c*_*L*_ = 0.01 mM) LiCl solution buffered with HEPES salt and an EDTA chelating agent (pH = 8.15). The aqueous stream of charged liposomes was pumped in the middle inlet channels of the upstream junction, whereas the same low salt concentration solution without liposomes was injected in the inner channel of the upstream junction. Finally, a high salt concentration (*c*_*H*_ = 6.65 mM) of LiCl solution, also buffered with HEPES salt and EDTA, was pumped in the outer inlet channels of the downstream junction. The fluorescence intensity profiles along the transverse direction are shown in Figure 6a. It can be observed that charged liposomes display behavior similar to the one observed for polystyrene nanoparticles. Upon imposition of a salt gradient (red curves), peaks of accumulated liposomes form and converge to the center of the device (|*x*|/*w*_*m*_ ≤ 0.2) and away from the colloidal bulk streams (|*x*|/*w*_*m*_ > 0.2). Conversely, when the salt concentration is the same throughout the device (blue curves), liposome peaks do not form and no particles migrate toward the central region of the channel. Note that for liposomes, a higher salt contrast (*c*_*H*_/*c*_*L*_ = 333) was adopted in comparison to the one applied in the experiments with carboxylated polystyrene nanoparticles (*c*_*H*_/*c*_*L*_ = 100). Indeed, in agreement with previous studies,^46,67^ the migration speed of liposomes under salt concentration gradients is typically smaller than the one of polystyrene nanoparticles with comparable size and zeta potential, therefore higher salt contrasts are required. This is likely due to the soft and water-permeable nature of the liposomes that may trigger additional migration mechanisms, such as osmophoresis,^68^ or affect the diffusiophoresis response through membrane permeability,^69,70^ membrane viscosity, and vesicle shape deformations. To confirm that the imposed salt contrast did not affect the liposome stability and size in our experiments, a liposome solution in low salt concentration (*c*_*L*_) buffer was analyzed via dynamic light scattering before and after dilution in high salt concentration (*c*_*H*_) buffer, resulting in no detectable changes in the particle size distribution. Interestingly, the inset in Figure 6a shows that when using zwitterionic DPPC liposomes (ζ = +10 mV) instead of negatively charged DPPC-DOPS liposomes, no focusing effect is observed under a salinity gradient. This observation is consistent with our physical interpretation of the particle focusing phenomenon. Indeed, DPPC liposomes in the buffer solution carry a very weak positive charge and, therefore, do not migrate by diffusiophoresis toward the top and bottom nonzero charged walls of the channel. In the absence of particle accumulation nearby the walls, the diffusio-osmosis flows at the walls do not affect the colloid distribution in the device and no particles are directed toward the center of the channel (|*x*|/*w*_*m*_ ≤ 0.2). Consequently, this finding could be exploited for the microfluidic separation of liposomes based on their surface charge. Indeed, for a mixture of negatively charged liposomes and zwitterionic lipid vesicles, only the charged liposomes will migrate toward the central region of the microchannel, whereas the trajectories of the zwitterionic lipid vesicles will not be affected by the salt concentration gradient and they will keep clear of the central region of the channel.

In another set of experiments, we investigated the effect of the lipid composition of the liposome membrane on particle focusing. Specifically, we adjusted the zeta potential of the liposomes by varying the amount of charged lipid content in the membrane composition, and we controlled the viscosity/fluidity of the membrane by using lipid mixtures with a fluid/gel phase transition temperature either above or below room temperature. Since altering the zeta potential of the liposomes significantly affects their diffusiophoresis mobility, it should be possible to correlate the charged lipid content with the intensity of the focusing effect in the device. On the other hand, it is known that the diffusiophoresis speed depends also on the Newtonian and Maxwell stress balance at the particle surface,^71^ which in turn depends on the viscosity of the membrane. However, to date, the role of membrane viscosity on the diffusiophoresis of lipid vesicles or living cells has yet to be explored. To carry out this analysis, we considered six different liposome populations at 1,2 and 10% DOPS (charged lipid) mole fraction added to zwitterionic lipids, either 1,2-dioleoyl-*sn*-glycero-3-phosphocholine (DOPC) for a disordered fluid phase membrane or (DPPC) for an ordered gel phase membrane. Three independent samples were produced for each liposome population, and for each sample, both the zeta potential and the corresponding focusing parameter were determined. All liposome populations had similar size (*d* = 166 ± 4 nm) and polydispersity index. Figure 6b and Figure 6c show how the focusing parameter *I* is positively correlated to the particle zeta potential and the DOPS (charged lipid) molar fraction. Furthermore, for a given DOPS concentration, there is no statistically significant difference between the focusing parameters observed for DOPC-DOPS fluid-like membrane vesicles (blue bars/symbols) and for DPPC-DOPS gel-like membrane vesicles (red bars/symbols), thereby showing the irrelevance of the liposome membrane viscosity for the diffusiophoresis transport under the examined conditions. The linear regression (dashed line) between liposome zeta potential and focusing parameter is given by
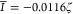
3with ζ expressed in millivolts. The regression line has a slope of 0.0116 with 95% confidence intervals of 0.0109 and 0.0124 for the lower and upper bounds, respectively. Equation 3 can be used as a calibration curve for the microfluidic detection of the zeta potential of similarly sized particles, whereby the focusing parameter *I̅* is measured experimentally to quantify the zeta potential.

Furthermore, we conducted an electrophoretic light scattering analysis to correlate the zeta potential of DOPC-DOPS vesicles with the DOPS (anionic lipid) content in the membrane (Figure 6d). As the DOPS molar fraction increases, the magnitude of the zeta potential of liposomes increases, and eventually it plateaus at ca. 8–10% DOPS content. Beyond this point, adding more DOPS to the membrane does not affect the zeta potential, due to the formation of a charged condensed layer of Na^+^ counterions around the outer leaflet of the liposomes.^72^ On the other hand, the zeta potential is highly sensitive to the DOPS content at low anionic lipid concentrations (≤3%). This suggests a strategy for the application of the double-junction device for the quantification of small amounts of DOPS lipids in the outer leaflet membranes of liposomes. Indeed, for low anionic lipid concentrations (≤3%), the relation between zeta potential ζ (expressed in millivolts) and the DOPS molar fraction *x*_DOPS_ (expressed in percentage values) can be well approximated by the following linear relationship, plotted as a dashed line in Figure 6d,
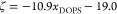
4By combining eq 3 and eq 4, it follows
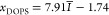
5which allows one to estimate the DOPS lipid molar fraction from the experimental measurements of the focusing parameter *I̅*. To conclude, eq 3 and eq 5 show how the double-junction device can be used effectively for the quantitative estimation of both the zeta potential and the DOPS lipid content of the outer leaflet of liposome membranes.

## Conclusions

We described a physical mechanism where nanoparticle diffusiophoresis and diffusio-osmosis flows along the channel walls are closely intertwined, leading to a strong transverse focusing of nanoparticles in a single Ψ-junction microchannel under continuous and steady axial flow conditions. Parallel electrolyte streams are merged at the junction of the device to generate a chemical gradient in both the transverse and vertical directions. As a result, the particles first migrate vertically by diffusiophoresis from the bulk toward the top and bottom walls and subsequently undergo a transverse horizontal migration along these walls driven by diffusio-osmosis. The coupling of diffusiophoresis and diffusio-osmosis along two perpendicular directions allows us to take advantage of the relatively weak diffusio-osmotic slip velocities in a pressure-driven microfluidic flow without resorting to dead-end or nanosized channels. Indeed, here it is the vertical diffusiophoretic migration that confines the particles near the charged walls, where the effects of diffusio-osmotic flows are the most intense. Consequently, one can avoid the most common drawbacks associated with dead-end pores or nanoconfined channels, such as device clogging due to particle accumulation or aggregation, costly device fabrication procedures, and difficult recovery of the colloidal sample. By observing the device via epi-fluorescence microscopy, two accumulation peaks are formed and their separation distance decreases with the distance from the channel inlet, eventually reaching a plateau value that depends on the size and ζ-potential of the colloids. Those peaks are of an intrinsically different nature of those observed in previous works,^37^ the dynamics of which was exclusively driven by diffusiophoresis.

We also showcased the exploitation of this mechanism for the continuous separation and characterization of colloidal particles. A proof-of-concept double-junction device was developed and used for the accurate measurements of the diameter of colloidal beads with the same ζ-potential, based on the measurement of an ad-hoc, purposely defined, focusing parameter *I̅*. We also demonstrated how the dependence of the transverse drift dynamics of the accumulation peaks on the colloid size can be further leveraged to separate large particles (1.098 μm) from smaller ones (36 nm) in a bimodal mixture. Moreover, the setup is proven to allow for a reasonable assessment of the ζ-potential of the same-sized particles through the measurement of the focusing parameter *I̅*. Based on this latter principle, we eventually showed how, in the case of liposomes, the measurement of the ζ-potential can be used to assess the chemical composition of the membrane (here, the molar fraction of the charged DOPS lipid), at least in the low concentration limit. Note that, while previous studies allowed only for batch measurements of particles located in dead-end pores or open nanochannels, our double-junction device enables the online, continuous and high-throughput characterization of particles directly within the colloid stream. This also facilitates the recollection of the analyzed sample for further off-chip downstream analysis. Finally, we showed how, under the examined experimental conditions, the fluid-like or gel-like states of the membrane, and thus the membrane viscosity, do not affect the diffusiophoretic response of the liposomes.

We envisage that this study will lead to alternative routes for exploiting diffusiophoresis and diffusio-osmosis for the microfluidic manipulation and characterization of both synthetic and natural particles. Potential biooriented applications of our microfluidic devices include the preconcentration, sorting, sensing, and analysis of biological entities, such as liposomes, extracellular vesicles, and bacteria. By relying on the chemical energies of the electrolyte solutions rather than on external energy sources and by adopting cheap and easy-to-fabricate microfluidic chips, the proposed particle manipulation and characterization strategies naturally lend themselves to the development of portable, cost-effective, point-of-need microdevices for chemical and biochemical analysis, diagnostics, drug screening, and drug delivery applications.

## Methods

### Materials

Invitrogen FluoSpheres, carboxylate-modified nanoparticles, red fluorescent (580/605), and 2% solids were purchased from ThermoFisher scientific at various sizes (20,100,200,500,1000 nm). In addition, Invitrogen FluoSpheres, yellow-green fluorescent (505/515), 2% solid, and 20 nm carboxylate-modified nanoparticles were also purchased for separation experiments from the same supplier. Lithium chloride salt (LiCl, 99%) used for diffusiophoresis experiments was purchased from Acros Organics. Aqueous liposome solutions were prepared with buffer salt, HEPES and chelating agent EDTA purchased from Sigma-Aldrich. Lipophilic dye, 3,3′-dioctadecyloxacarbocyanine perchlorate (DiO_*c*_18__) (used for staining liposomes) and chloroform (99%) (used for preparing lipid films) were purchased from Sigma-Aldrich. RTV 615 polydimethylsiloxane used for the fabrication of microfluidic devices was purchased from Techsil, UK. The phospholipids 1,2-dioleoyl-*sn*-glycero-3-phosphocholine (DOPC), 1,2-dipalmitoyl-*sn*-glycero-3-phosphocholine (DPPC), and 1,2-dioleoyl-*sn*-glycero-3-phospho-l-serine (DOPS) were purchased from Avanti Polar Lipids. Deionized water (18.2 MΩ cm) produced from an ultrapure milli-Q grade purification system (Millipore, USA) was used to prepare all aqueous solutions.

### Fabrication and Operation of the Microfluidic Devices

Standard photolithography and soft-lithography techniques were employed in manufacturing the microfluidic devices. Briefly, the CAD drawings of both one- and two-junction devices were printed on a photomask film (Micro Lithography Services, UK) and subsequently used to produce an SU-8 master mold of ca. 50 μm thickness on a silicon wafer (Inseto, UK). Imprinted polydimethylsiloxane (PDMS) channels are then made via replica molding by heating a PDMS:curing agent (9:1) mixture on top of the SU-8 master mold. The channels are then irreversibly bonded to a microscope slide by using a plasma cleaner (Harrick Plasma, UK) at 18W power for 1 min. All devices have a nominal depth of 50 μm and a total channel width of *w* = 400 μm. For the single junction chip, the widths of the main channel and the inner inlet channel are *w* = 400 μm and *w*_*i*_ = 200 μm, respectively, whereas the widths of the outer inlet channels are (*w* – *w*_*i*_)/2 = 100 μm. The flow rate in the inner channel (3.65 μL/min) matches the sum of the flow rates in the outer channels so that the inner and outer streams have the same average velocity and the width of inner stream is not altered by hydrodynamic focusing or broadening effects. For the double-junction device, the width of the main channel is *w* = 400 μm. The widths of the middle channel and the outer inlet channels, meeting at the wider (downstream) junction, are *w*_*m*_ = 250 μm and (*w* – *w*_*m*_)/2 = 75 μm, respectively. The widths of the inner and middle inlet channels, meeting at the narrower (upstream) junction, are *w*_*i*_ = 100 μm and (*w*_*m*_ – *w*_*i*_)/2 = 75 μm, respectively. The flow rate in the inner channel (3.65 μL/min) is equal to the sum of the flow rates in the middle inlet channels, which in turn matches the sum of the flow rates in the outer inlet channels. Therefore, the average velocity of the middle and outer streams is slightly lower than the average velocity of the inner stream. As detailed in Supporting Information, this leads to a weak hydrodynamic focusing of the two middle colloidal streams and a slight broadening of the inner particle-free stream. Aqueous solutions are injected into the device inlets by means of syringe pumps (Harvard, USA).

### Nanoparticle Sample Preparation

Carboxylate polystyrene particles (2% solids) were diluted in a 0.1 mM LiCl solution at a particle concentration of 0.002% (v/v) for the 1 μm particles and 0.02% (v/v) for particles of other diameters. Liposomes were prepared using standard thin film hydration^73^ and subsequent extrusion techniques.^74^ Briefly, lipids and lipophilic dye (DiO_*c*_18__) were dissolved in chloroform and mixed at appropriate ratios. The chloroform was evaporated by using a constant flow of N_2_. The dried lipid film was desiccated for a minimum of 3 h before hydration and dilution using a 0.01 mM LiCl + 5 μM EDTA + 5 μM HEPES solution, pH = 8.15. The liposome suspension was then vortexed at 1200 rpm for 60s and extruded 21 times through a 200 nm polycarbonate membrane filter (Avanti Polar Lipids, US) to maintain an average polydispersity index (PDI) of 0.1.

### Image Acquisition and Processing

An inverted optical microscope (TE300, Nikon) equipped with a 10x objective lens (0.25 NA) is used to image particle dynamics at different distances from the junction. A fluorescent lamp (CoolLED pE300) is used to excite the sample, which allows the collection of fluorescent intensity data via a CCD camera (Ximea MQ013MG-ON). Collected data are in the form of 1264 × 1016 px, 16 bit TIFF images. The depth of field of the microscope, i.e., the thickness of the slice region that is in acceptably sharp focus in the micrographs, can be estimated as^75^*n*_ind_ λ_em_/*NA*^2^ + *n*_ind_*e*/(*M*·*NA*) ≃ 10 μm, where *n*_ind_ = 1 is the refractive index of the objective immersion medium (air), λ_em_ = 510 nm is the nanoparticle emission wavelength, *e* = 4.8 μm is the pixel pitch of the CMOS camera, *NA* = 0.25 and *M* = 10 are the objective numerical aperture and magnification, respectively. The depth of the measurement volume is larger than the depth of field, since the fluorescence signal generated by out-of-focus particles also contributes to the formation of the image at the camera sensor. The epi-fluorescence micrographs were acquired by positioning the focal plane near the bottom (glass) wall. Experimental observations (Figure S3 in the Supporting Information) showed that the micrographs displayed the same fluorescence intensity when the focal plane was located at the top (PDMS) and bottom (glass) walls as well as at the midpoint along the channel depth of the device, confirming that the depth of the measurement volume is comparable to the channel depth. The location of the focal plane was established by focusing on colloidal particles permanently stuck on the bottom and top walls of the microchannel. The fluorescence intensity profiles were normalized by first subtracting the background noise and then dividing the fluorescence intensity of the bulk colloidal streams in the absence of a salinity gradient (∇*c* = 0). The background intensity level was calculated as the minimum intensity value of the fluorescence profiles obtained under no salinity gradient conditions.

A PicoQuant MicroTime 200 time-resolved confocal microscopy platform, built around an Olympus IX 73 microscope, was used to acquire confocal images in the (*x*-*y*) plane, as shown in Figure 1. A plan N 40× water immersion objective lens (0.65 NA) was used to take 1024 × 1024 px, 32 bit TIFF images. A monodirectional scanning pattern was used with a learning time of 5 s and a dwell time of 2 s. For confocal images, the fluorescence intensity was normalized by dividing by the average intensity of the bulk colloidal stream under no salt concentration gradient. All fluorescent images shown in the figures were processed using ImageJ (contrast enhancement, LUT color change).

### Particle Characterization

All size and zeta potential measurements were performed using a ZetaSizer nano ZS (Malvern Panalytical) at 25 °C. All samples were analyzed a minimum of 3 times using the monodomal measurement mode for a monodisperse single population of particles. The instrument provided the zeta potential values calculated according to Smoluchowski’s theory for which the Debye length is much smaller than the particle size. The zeta potential values were corrected to account for the finite size of the Debye length according to Henry’s model.^76^

### Numerical Methods

Numerical simulations were performed in Comsol Multiphysics according to the procedures detailed in our previous work.^38^ Briefly, the 3D computational domain consisted of a rectangular channel of width 2*w*_*i*_ = 400 μm, depth *h* = 52 μm, and length 25*w*_*i*_ + 5*h*. The hydrodynamic velocity, ***u***, pressure *p*, salt concentration *c*, and particle concentration *n* were calculated by solving the steady-state Navier–Stokes equation and the advection-diffusion equations for *c* and *n*. At the channel inlet, the boundary condition for the velocity field was ***u*** = ***u***_inlet_, with ***u***_inlet_ being the fully developed velocity field at a cross section of the rectangular channel perpendicular to the flow direction and with average velocity *U*_0_. The boundary conditions at the channel inlet for the salt and particle concentration fields were *c* = *c*_*H*_ and *n* = 0 for the outer flow region (i.e., |*x*| > *w*_*i*_/2) and *c* = *c*_*L*_ and *n* = *n*_0_ for the inner flow region (i.e., |*x*|≤ *w*_*i*_/2). At the channel outlet, the zero normal gradient boundary conditions for the pressure, salt, and particle concentrations were imposed. At the remaining walls, the slip boundary condition ***u*** = −Γ_DO_**∇**(ln *c*) was applied together with the zero flux condition for the salt and particle concentration fields. The channel outlet was located at 5 times the channel depth *h* from the cross section *z*/*w*_*i*_ = 25 to ensure that the boundary conditions at the channel outlet do not affect the fields near that section. The particle diffusiophoresis coefficient Γ_DP_ = 291 μm^2^/s was calculated according to the procedure detailed in our previous work,^38^ where the formula provided by Prieve and co-workers^47^ was used to account for the particle size effect on Γ_DP_. A diffusio-osmosis coefficient of Γ_DO_ = 1165 μm^2^/s was chosen for the channel walls to obtain a good quantitative match between experimental results and numerical predictions. As detailed in the Supporting Information, this value is within the expected range for glass and PDMS walls in a LiCl solution at a few millimolar concentrations and neutral pH.

## Data Availability

The data underlying this study are openly available on University College London repository doi.org/10.5522/04/23302169.

## Supporting Information Available

Results from control experiments in a single Ψ-junction device without a salt concentration gradient; results from experimental test in a single Ψ-junction device with colloidal particles dispersed in the higher salt concentration stream; epi-fluorescence micrographs for different locations of the objective’s focal plane in a Ψ-junction device under a salinity gradient; estimates of the diffusio-osmosis coefficient for the microchannel walls; analysis of the hydrodynamic focusing and broadening effects of streams in the double-junction device. (PDF)
