# Supplementary material for: Continuous Manipulation and Characterization of Colloidal Beads and Liposomes via Diffusiophoresis in Single- and Double-Junction Microchannels
Source: ACS Nano. 2023 Jul 17;17(15):14644–57. doi: 10.1021/acsnano.3c02154 (PMC10416570; doi:10.1021/acsnano.3c02154)
Supplement: Supplementary file 1 — nn3c02154_si_001.pdf [file nn3c02154_si_001.pdf]

Continuous manipulation and characterization of colloidal beads and liposomes via diffusiophoresis in single- and double-junction microchannels

Adnan Chakra,[†,‡] Naval Singh,[¶] Goran T. Vladisavljević,[†] François Nadal,[§] Cécile Cottin-Bizonne,[‖] Christophe Pirat,[‖] and Guido Bolognesi[*,‡,†]

†*Department of Chemical Engineering, Loughborough University, Loughborough, LE11 3TU, United Kingdom*

‡*Department of Chemistry, University College London, London, WC1H 0AJ, United Kingdom*

¶*Manchester Centre for Nonlinear Dynamics, Department of Physics and Astronomy, University of Manchester, Manchester M13 9PL, United Kingdom*

§*Commissariat à l'Énergie Atomique, BP2, 33114, Le Barp, France*

‖*Institut Lumière Matière, UMR5306 Université Claude Bernard Lyon 1 - CNRS, Université de Lyon, Villeurbanne Cedex, 69622, France*

E-mail: g.bolognesi@ucl.ac.uk

Supporting Information

Control Experiment in a Single Ψ-junction Chip

A control experiment with no salt concentration gradient was conducted by using the same Ψ-junction microchip in the main text. A colloidal suspension at low salt concentration (c_L) is injected in the inner channel, whereas an aqueous solution with an identical level of salinity (c_L) is pumped in the outer channels (Figure S1a). In the absence of a chemical gradient, the colloids remain homogeneously distributed within the inner region of the channel and the colloidal band maintains a constant width downstream of the junction (Figure S1b). No peak formation and subsequent convergence are visible and the particle distribution profile along the channel depth remains uniform (Figure S1c). This is because no salinity gradient exists in any direction. Consequently, electrokinetic phenomena such as diffusiophoresis and diffusiosmosis do not occur in the system. Furthermore, the flow is characterized by a high Peclet number, $Pe = U_0\,w/D_c \simeq 10^6$, where D_c is the particle diffusivity given by $D_c = k_bT/6\pi\mu a = 2.4 \times 10^{-12}\,\mathrm{m^2/s}$ with $\mu = 0.9 \times 10^{-3}\,\mathrm{Pa{\cdot}s}$ as the solution viscosity and $a = 108$ nm as the particle radius. Therefore, particle diffusion is negligible compared to

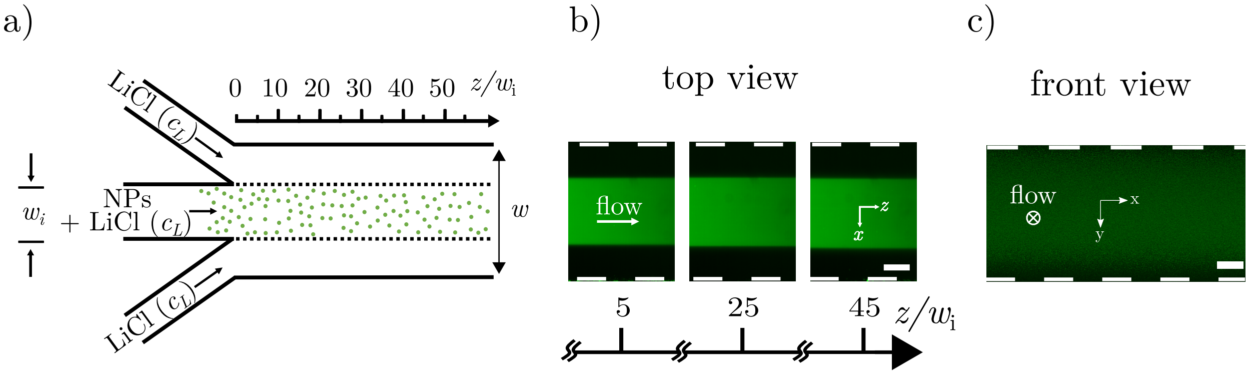

Figure S1: Particle dynamics under no salinity gradient conditions $\nabla c = 0$. (a) Schematic diagram of a single Ψ-junction microchip, where low salt concentration is injected in all channels with carboxylate polystyrene nanoparticles (NPs) present in the central channel only. (b) Top view epi-fluorescence images taken at different distances downstream of the junction for the flow configuration shown in (a), scale bar = 75 μm. (c) Confocal image taken at $z/w_i = 25$ for the flow configuration shown in (a), scale bar = 10 μm.

convective transport, and since both inertia and gravity effects are also negligible, the colloids behave as passive tracers.

Salinity Conditions of Nanoparticle Solution for Peak Formation

We find that the peak formation due to the reported focusing effect does not occur when the carboxylate polystyrene nanoparticles are dispersed in the high salt (c_H = 10 mM) solution in the outer channels and not in the low salt (c_L = 0.1 mM) solution in the inner channel (Figure S2a). Consequently, this led to the development of the double Ψ-junction microchip for nanoparticle fractioning to target advanced applications. Moreover, we observe

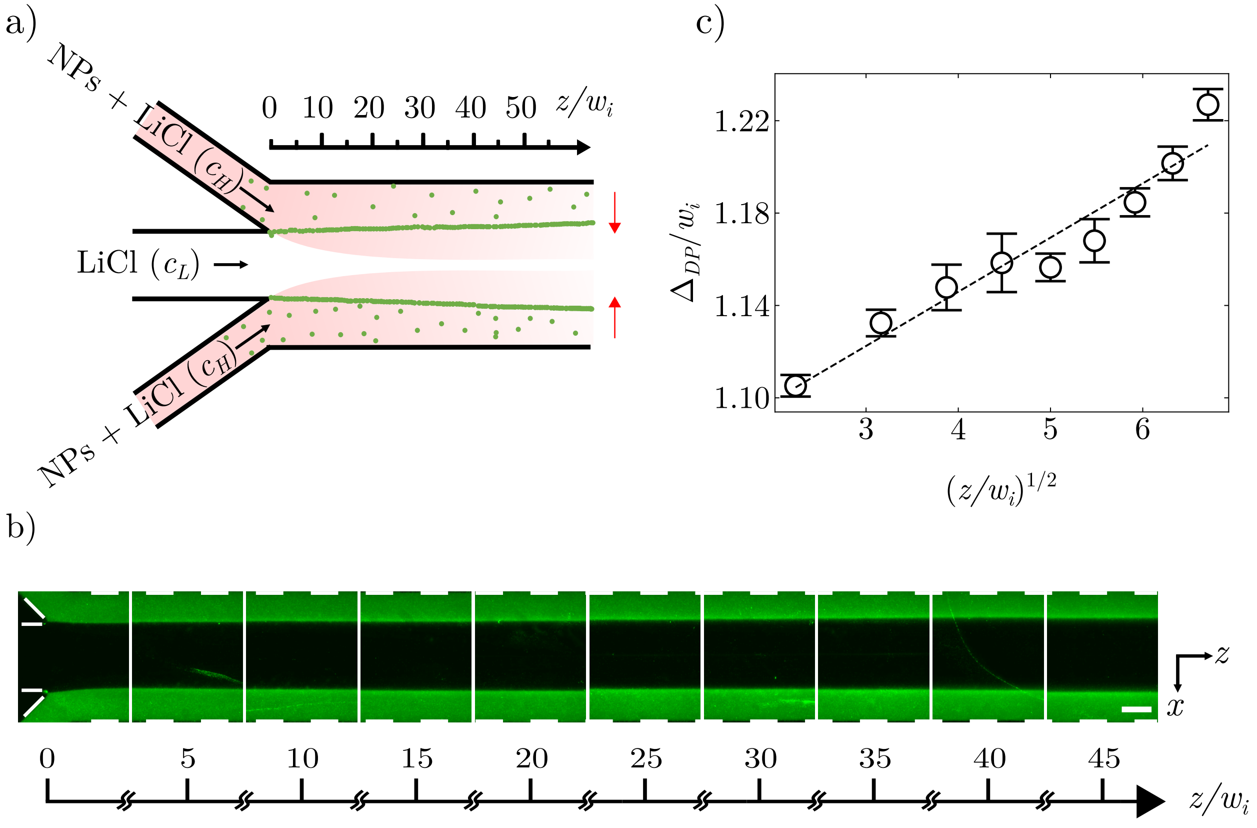

Figure S2: Absence of the hereby reported focusing mechanisms when colloids are in high salinity. (a) Schematic diagram of a single Ψ-junction microchip, where low salt is injected in the inner channel and high salt with colloids is injected in the outer channels. (b) Epi-fluorescence images taken at different distances downstream of the junction for the flow configuration in (a), scale bar = 75 μm. (c) The dynamics of particle migration against the square root of the longitudinal distance downstream of the junction.

a previously reported particle accumulation effect that has been outlined in the main text. Figure S2c substantiates this, as accumulated particles diverge towards higher salinity regions at a $\sqrt{z}$ trend downstream of the junction.

Fluorescent Intensity Micrographs at Different Focal Planes in a Single Ψ-junction Chip

Figure S3 shows the fluorescence intensity profiles along the transverse direction in a single junction device in the presence of a salt concentration gradient for a focal plane of the microscope objective located nearby (i) the top PDMS wall, (ii) the midpoint along the channel depth and (iii) the bottom glass wall. The location of the focal plane was established by focusing on colloidal particles permanently stuck on the bottom and top walls of the microchannel. From these profiles it can be seen that there is no considerable difference in fluorescence intensity and peak convergence. Therefore, the results generated from epi-fluorescence micrographs captured by our fluorescence microscope are independent of the focal plane location.

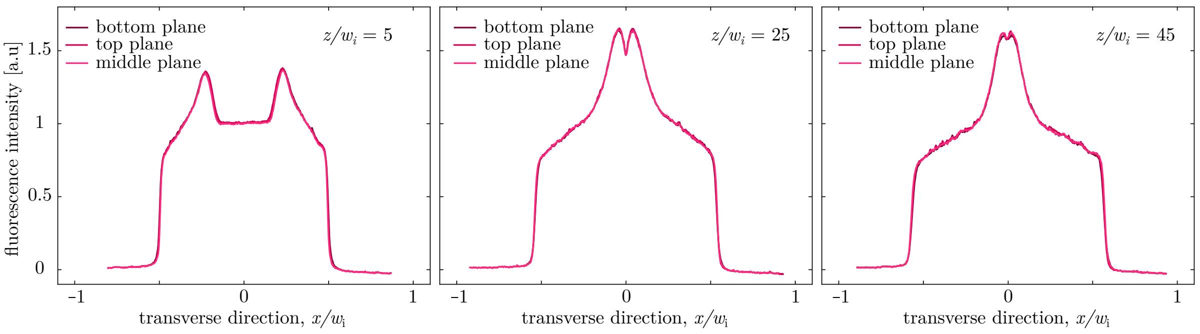

Figure S3: Fluorescence intensity profiles along the transverse x-direction at increasing distances z/w_i downstream of the junction in the presence of a salt concentration gradient. The experimental conditions (e.g. electrolyte concentrations, particle type, flow rates, etc.) are the same ones adopted for the experimental results reported in Figure 1. The profiles were generated from epi-fluorescence micrographs with the focal planes located (i) nearby the glass wall (bottom plane), (ii) the midpoint along the channel depth (middle plane), and (iii) the PDMS wall (top plane).

Estimate of Diffusioosmosis Coefficient for Glass and PDMS Walls

The diffusioosmosis (DO) coefficient Γ_{DO} can be calculated as

$$\Gamma_{\text{DO}} = \frac{\varepsilon}{2\eta}\left(\frac{k_bT}{Ze}\right)^2\left[2\beta\bar{\zeta} - 4\ln(1-\gamma^2)\right] \tag{S1}$$

where $\gamma = \tanh(\bar{\zeta}/4)$ and $\beta = \frac{D_+ - D_-}{D_+ + D_-}$, ε is the absolute permittivity of the surrounding liquid medium, η is the medium viscosity, k_bT is the thermal energy, Z is the ion valence, e is the elementary charge, $\bar{\zeta} = \frac{Ze\zeta}{k_bT}$ is the adimensionalised zeta potential and D_+ and D_- are the diffusivities of cations and anions, respectively. For LiCl aqueous solution at 25°C, $\varepsilon = 85.8$, $\eta = 0.9\,\text{Pa}\,\text{s}$, $D_+ = 1.026 \times 10^{-9}\,\text{m}^2/\text{s}$ and $D_- = 1.964 \times 10^{-9}\,\text{m}^2/\text{s}$.

Kirby and Hasselbrink Jr developed[1,2] an empirical model based on literature experimental data that can predict the zeta potential of glass and native (untreated) PDMS surfaces under varying salt and pH conditions. The model applies to indifferent univalent electrolytes, such as Na and K, for which the surface-ion binding is independent on the ionic strength of the solution. By assuming that the surface charge density does not depend on the counterion type and concentration, the zeta potential of a substrate can be approximated as $\zeta\,[\text{mV}] = -a_1 \log c\,[\text{M}]$, where c is the electrolyte concentration. For glass (silica) surfaces, $a_1 \simeq 2 + 7\cdot(\text{pH} - 3)$, thus, at neutral condition (pH=7), $a_1 = 30$ and the model predicts $\zeta = -90\,\text{mV}$ for $c = 1\,\text{mM}$. It is now reasonable to expect that the model assumptions are valid also for Li counterions in the millimolar concentration range and thus, according to Eq. (S1), one can predict a Γ_{DO} value of 1380 $\mu\text{m}^2/\text{s}$ for LiCl solutions. For native (untreated) PDMS with solution in the pH range between 6.5 and 7, $a_1 \simeq -27\,\text{mV}$ and, thus, the predicted value of the zeta potential is $\zeta = -81\,\text{mV}$ for $c = 1\,\text{mM}$. This leads to a calculated Γ_{DO} value of 1180 $\mu\text{m}^2/\text{s}$ for LiCl solutions. Therefore, the numerical value used in the numerical simulations for the diffusioosmosis coefficient ($\Gamma_{\text{DO}} = 1165\,\mu\text{m}^2/\text{s}$) is within the expected range for glass and PDMS walls in a LiCl solution at a few millimolar concentration and neutral pH.

Hydrodynamic Focusing and Broadening of Streams in Double-junction Device

In a Ψ-junction, a mismatch between the average velocity of the inner and outer streams leads to the hydrodynamic focusing or broadening of the inner stream. Such hydrodynamic effects can be estimated as follows. Let us consider a Ψ-junction (Figure S4) consisting of an inner inlet channel of width w_{in}, two symmetric outer inlet channels of width w_{out} and an outlet (main) channel of width w_c. Let us denote w_{in}^{∞} as the final width achieved by the inner stream after that the flow is fully developed downstream of the junction. The predicted value of w_{in}^{∞} is given by the following relation[3]

$$\frac{w_{in}^{\infty}}{w_c} = \frac{Q_{in}}{Q_{out} + Q_{out}} \tag{S2}$$

where Q_{in} is the flow rate of the inner stream injected into the inlet channel and Q_{out} is the total rate of the outer flow, which is equally split between the two outer inlet channels. Eq. (S2) is valid when, in the outlet channel, the average velocity of the inner stream is equal to the average velocity of the entire flow in the channel – a condition that holds for large values of the channel depth/width ratios.[3] By applying Eq. (S2) to the first (upstream)

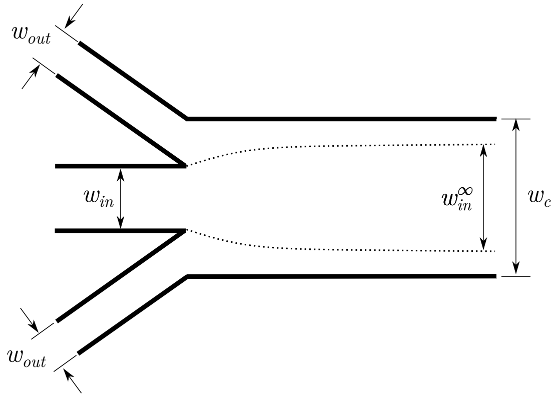

Figure S4: Hydrodynamic broadening effect at a Ψ-junction.

Table S1: Values of the parameters used in Eq.(S2) to estimate the effects of hydrodynamic broadening in the upstream and downstream junctions of the double-junction device.

**Parameters**	**Upstream junct.**	**Downstream junct.**
w_{in}	$w_i = 100\,\mu\text{m}$	$w_m = 250\,\mu\text{m}$
w_c	$w_m = 250\,\mu\text{m}$	$w = 400\,\mu\text{m}$
Q_{in}	3.65 μL/min	3.65 μL/min
Q_{out}	3.65 μL/min	3.65 μL/min

junction of the double-junction device (see parameters in Table S1), it is found that the final width of the inner stream, $w_{in}^{\infty} = 125\,\mu\text{m}$, is larger than the initial width, $w_{in} = 100\,\mu\text{m}$. Consequently, the inner (particle-free) stream expands slightly, whereas the widths of two middle colloidal streams are slightly reduced, going from $(w_m - w_{in})/2 = 75\,\mu\text{m}$ in the middle inlet channels down to $(w_m - w_{in}^{\infty})/2 = 62.5\,\mu\text{m}$ in the middle channel. By applying Eq. (S2) to the second (downstream) junction (see parameters in Table S1), it is found that the final width of the flow composed of the inner and middle streams, $w_{in}^{\infty} = 267\,\mu\text{m}$, is larger than its initial value, $w_{in} = 250\,\mu\text{m}$. As a result, the width of the two middle colloidal streams increases slightly after the second junction, going from 62.5 μm in the middle channel up to $62.5 \times \frac{w_{in}^{\infty}}{w_{in}} = 66.8\,\mu\text{m}$ in the main channel. Similarly, the width of the inner (particle-free) stream increases from 125 μm in the middle channel to $125 \times \frac{w_{in}^{\infty}}{w_{in}} = 133.5\,\mu\text{m}$ in the main channel. Note that the hydrodynamic focusing and broadening effects are relatively fast and the final widths of the streams are rapidly established at short distances from the junctions. Consequently, the advective transport of particles induced by the hydrodynamic focusing/broadening of the streams is decoupled from the particle dynamics induced by diffusiophoresis and diffusioosmosis. Indeed, all epi-fluorescence intensity profiles reported in the manuscript are taken at least 1 mm downstream of the junction, which corresponds to 20 times the channel depth. At such a distance, the width of the streams and the dynamics of the particles are no longer affected by hydrodynamic focusing/broadening effects. In other

words, the final widths of the inner and middle streams serve as the initial configuration to which the reported solute-driven particle focusing mechanism applies. This interpretation is confirmed by the fluorescence intensity profiles in Figure 3c, which show that, in the absence of a salt concentration gradient, the width of the inner (particle-free) stream remains constant for $z/w_m \geq 4$. Interestingly, the hydrodynamic broadening of the inner (particle-free) stream is advantageous for the proposed applications of the double-junction device. In fact, the slight broadening of the particle-free flow leads to a further increase in the distance between the particle accumulation peaks, that converge towards the central region of the device, and the two bulk middle colloidal streams on the sides of the main channel. This facilitates further the decoupling of the fluorescent signals collected from the accumulation peaks and the bulk colloidal streams. It is important to note that the hydrodynamic broadening of the inner stream does not affect the working principle and application of the double-junction device and similar particle dynamics could be observed if the flow rates were adjusted to prevent any hydrodynamic broadening or focusing effect.

References

1. Kirby, B. J.; Hasselbrink Jr, E. F. Zeta Potential of Microfluidic Substrates: 1. Theory, Experimental Techniques, and Effects on Separations. *Electrophoresis* **2004**, *25*, 187–202.

2. Kirby, B. J.; Hasselbrink Jr, E. F. Zeta Potential of Microfluidic Substrates: 2. Data for Polymers. *Electrophoresis* **2004**, *25*, 203–213.

3. Lee, G.-B.; Chang, C.-C.; Huang, S.-B.; Yang, R.-J. The Hydrodynamic Focusing Effect inside Rectangular Microchannels. *J. Micromech. Microeng.* **2006**, *16*, 1024.
